# Chlorogenic Acid Improves Intestinal Health in Largemouth Bass (*Micropterus salmoides*) by Enhancing Antioxidant Defense, Reducing Inflammatory Responses, and Modulating the Gut Microbiota

**DOI:** 10.3390/ani16111668

**Published:** 2026-05-29

**Authors:** Qin Zhang, Lan Li, Dehong Lan, Miao Zhou, Ziyang Yuan, Tong Tong, Yongqiang Liu, Zhichang He, Zhongbao Guo, Weiguang Kong

**Affiliations:** 1 Guangxi Key Laboratory for Polysaccharide Materials and Modifications, Guangxi Marine Microbial Resources Industrialization Engineering Technology Research Center, School of Marine Sciences and Biotechnology, Guangxi Minzu University, 158 University West Road, Nanning 530008, China; zhangqin@gxmzu.edu.cn (Q.Z.); lilan@stu.gxmzu.edu.cn (L.L.); landehong@stu.gxmzu.edu.cn (D.L.); zhoumiao@stu.gxmzu.edu.cn (M.Z.); yuanziyang@stu.gxmzu.edu.cn (Z.Y.); tongtong@gxmzu.edu.cn (T.T.); 2 Guangxi Key Laboratory for Aquatic Genetic Breeding and Healthy Aquaculture, Guangxi Academy of Fishery Science, 8 Qingshan Road, Nanning 530021, China; guozhongbaono1@163.com; 3 Key Laboratory of Breeding Biotechnology and Sustainable Aquaculture, Institute of Hydrobiology, Chinese Academy of Sciences, 7 Donghu South Road, Wuhan 430072, China; kongweiguang@ihb.ac.cn

**Keywords:** feed additive, plant polyphenols, redox homeostasis, intestinal histomorphology, aquaculture nutrition

## Abstract

Intestinal oxidative imbalance, inflammatory activation, and microbial dysbiosis are increasingly recognized as major factors affecting the health status of intensively cultured largemouth bass (*Micropterus salmoides*). Chlorogenic acid (CGA) is a plant-derived polyphenolic compound that is well known for its antioxidant and anti-inflammatory properties. Although its inclusion as a dietary supplement in aquafeeds has garnered increasing interest, its effects on intestinal health in largemouth bass under normal rearing conditions remain largely unknown. Hence, the present investigation was designed to elucidate how incrementally elevated dietary CGA concentrations modulate oxidative capacity, inflammatory gene expression, mucosal histological features, and enteric microbiota structure in juvenile largemouth bass. In this study, largemouth bass were fed diets containing 0, 200, 400, or 600 mg/kg CGA for 70 days, and we evaluated intestinal antioxidant capacity, inflammation-related transcriptional responses, histomorphology, and gut microbiota were assessed. The results demonstrated that CGA supplementation significantly raised intestinal total antioxidant capacity as well as the activities of superoxide dismutase, catalase, glutathione peroxidase, and glutathione reductase, while substantially decreasing malondialdehyde levels (*p* < 0.05). The transcription levels of *cat*, *gsh-px*, *gst*, *sod*, *nrf2*, and *ucp2* were markedly upregulated, whereas *keap1* expression was downregulated (*p* < 0.05). CGA supplementation also reduced the transcription levels of several proinflammatory genes, including *il-1β*, *il-6*, *il-8*, *tnf-α*, *nf-κb*, *p50*, *map3k*, *jak2*, as well as *stat3*, while significantly elevating *il-10* expression, in the 200 and 400 mg/kg groups, *tgf-β* expression was also lowered (*p* < 0.05). Histological examination revealed that CGA supplementation influenced intestinal morphology in a dose-dependent manner; the most consistent improvements in villus length, villus width, and muscularis thickness occurred at the 400 mg/kg level, whereas the 600 mg/kg level showed weaker effects in several parameters compared with the control. Furthermore, 16S rRNA sequencing indicated that CGA altered microbial diversity, community structure, and predicted functional profiles in the intestine. In summary, dietary CGA supplementation was associated with enhanced intestinal antioxidant capacity, modified inflammation-related transcriptional responses, changes in intestinal morphology, and shifts in the gut microbial community of largemouth bass. Within the tested dose range, the 400 mg/kg group exhibited relatively favorable responses in several measured indicators under the present experimental conditions.

## Simple Summary

The intestine is closely related to nutrient utilization and physiological condition in farmed fish. In this study, juvenile largemouth bass were fed diets supplemented with different levels of chlorogenic acid (CGA) for 70 days to evaluate its effects on intestinal antioxidant status, inflammation-related gene expression, intestinal morphology, and gut microbiota. The results revealed that dietary CGA supplementation increased antioxidant enzyme activities, decreased malondialdehyde levels, altered several inflammation-related transcripts, and improved certain intestinal morphological parameters. CGA also altered the diversity and composition of the gut microbial community. Relative to the control group, the 400 mg/kg treatment yielded superior antioxidant enzyme performance, increased villus length and breadth, and enriched gut bacterial diversity across all evaluated concentrations. The present data imply that CGA is capable of modulating intestinal physiological homeostasis in largemouth bass under routine farming conditions, although the defensive role of this compound during stress or infectious challenge still awaits experimental confirmation.

## 1. Introduction

In China’s aquaculture sector, the largemouth bass (*Micropterus salmoides*) ranks among the most commercially valuable freshwater fish species. Its widespread use derives from rapid growth, high market value, and strong consumer demand. As farming becomes more intensive and formulated feeds are increasingly relied upon, maintaining the health of cultured bass has become an urgent concern [1,2,3]. Earlier studies have shown that high stocking densities, alterations in dietary composition, and diverse environmental stressors can exacerbate oxidative stress, disrupt immune homeostasis, and increase disease susceptibility, thereby ultimately reducing production efficiency and the stability of aquaculture systems [4,5,6]. Recently, growing attention has been given to intestinal and hepatic disorders occurring during largemouth bass culture. Consequently, there is practical significance in developing safe feed additives that can support intestinal antioxidant capacity, immune-related responses, and tissue integrity in farmed fish.

In teleosts, the gut serves as both the primary site for digestion and nutrient uptake and a critical protective organ involved in immune defense and internal homeostasis [7]. Its health status relies on the integrated regulation of redox balance, inflammatory activity, tissue integrity, and the intestinal microbial community [8]. Under intensive rearing conditions, factors such as diet, environmental disturbances, and pathogen challenges can trigger oxidative stress and inflammation in the intestinal tissue, compromise epithelial structure and barrier function, and disturb microbial equilibrium, which in turn may affect nutrient utilization, immune function, and overall health [9,10,11]. For this reason, screening and evaluating functional feed additives from multiple perspectives—including antioxidant support, inflammation modulation, structural protection, and microbial balance—have become major research priorities in aquaculture nutrition and fish health [8,12]. Among the available candidates, plant-derived bioactive compounds have drawn considerable interest because of their natural origin, relatively high safety, and broad regulatory potential, and they are viewed as promising agents for improving intestinal condition and stress tolerance in aquatic animals [13].

Chlorogenic acid (CGA) is a naturally plant compound found at high levels in coffee beans, honeysuckle, Eucommia ulmoides, as well as chrysanthemum [14]. A growing body of evidence indicates that CGA possesses antioxidant, anti-inflammatory, and gut-protective properties [15,16]. In various animal models, CGA has been reported to elevate antioxidant enzyme activities, decrease lipid peroxidation, and modulate gene expression or factors associated with inflammatory pathways [17,18]. Evidence also suggests that CGA may influence intestinal morphology and gut microbiota composition [19]. In aquatic species, dietary CGA has been demonstrated to improve antioxidant status and survival in Pacific white shrimp (*Litopenaeus vannamei*) under combined stress [20]. In some fish, such as grass carp (*Ctenopharyngodon idella*), CGA supplementation has been linked to better growth, stronger antioxidant capacity, and improved health status [21]. Collectively, these findings indicate that CGA holds good potential as a plant-derived functional additive in aquaculture.

Although CGA has been examined in several aquatic species, most previous studies have focused on its effects on growth performance, lipid metabolism, antioxidant responses, and health-related indicators under dietary stress or challenge conditions. By contrast, information on how CGA influences baseline intestinal physiology in largemouth bass under normal rearing conditions is still lacking. In particular, the impact of graded dietary CGA supplementation on intestinal antioxidant capacity, inflammation-related transcriptional responses, tissue morphology, and gut microbial communities in this species remains unclear. To address this gap, the present study assessed whether dietary supplementation with 0, 200, 400, or 600 mg/kg CGA modulates intestinal antioxidant parameters, inflammation-related gene expression, histomorphology, and gut microbiota composition in juvenile largemouth bass raised under standard culture conditions. Based on the antioxidant and intestinal regulatory properties of CGA documented in previous studies, we hypothesized that appropriate dietary CGA supplementation would enhance intestinal antioxidant status, modulate signaling pathways related to inflammation and apoptosis, and alter the intestinal microbial composition in largemouth bass. We further hypothesized that these beneficial effects might be dose-dependent, with excessive supplementation leading to reduced physiological benefits.

## 2. Materials and Methods

### 2.1. Source of Chlorogenic Acid and Experimental Design

The chlorogenic acid (CGA) used in this study was purchased from Huazhong Haiwei (Beijing) Gene Technology Co., Ltd. (Beijing, China) and had a purity of 99%. The dietary inclusion levels of CGA were determined based on previous studies in aquatic animals [15,22,23], together with preliminary feeding observations conducted in this work. Four dietary treatments were established, containing 0 mg/kg CGA (G0, control), 200 mg/kg CGA (G1), 400 mg/kg CGA (G2), and 600 mg/kg CGA (G3), respectively.

### 2.2. Experimental Diet Preparation

The basal formulation without any CGA addition was used as the control diet (G0). For the G1, G2, and G3 groups, 200, 400, and 600 mg/kg of chlorogenic acid were respectively incorporated into the same basal formulation. The intended CGA concentrations in the diets were defined according to the experimental design. With the sole exception of the CGA supplementation level, all treatment diets possessed identical ingredient compositions and proximate nutritional profiles. The detailed composition and nutritional analysis of the basal diet are presented in Table 1.

To sustain isonitrogenous and isolipidic conditions across all treatments, the experimental feeds were formulated by integrating the requisite CGA dose while removing a corresponding wheat flour portion. Given the low inclusion levels (0.2–0.6 g/kg diet), this substitution was not expected to alter the proximate composition of the diets, which was subsequently confirmed by chemical analysis (Table 1). For diet preparation, dry ingredients were first milled and screened to obtain comparable particle size. According to the formulation, the main protein and carbohydrate ingredients—namely fish meal, soybean meal, soybean flour, wheat flour, and yeast powder—were weighed and then mixed until a visually uniform appearance was achieved. After that, lipid sources, mineral and vitamin premixes, choline chloride, calcium phosphate, and antioxidant were incorporated into the mixture. For the CGA-supplemented diets, the required amount of CGA was first premixed with a small portion of the basal feed powder and subsequently blended into the entire batch to enhance distribution homogeneity. The control diet was formulated following an identical protocol, differing only in the absence of CGA.

After adding deionized water to the mixed ingredients at a 50% (*w*/*w*) ratio, we pelleted the resulting mash to produce feeds of comparable size. The pellets were dried under cool, well-ventilated conditions until a constant weight was reached. After cooling, the diets were placed in labeled bags, sealed, and stored at −20 °C in the dark until feeding.

### 2.3. Rearing Management

The juvenile largemouth bass for this study were sourced from the Guangxi Improved Aquatic Seed Hainan Breeding Base (Ledong, Hainan, China). We subsequently performed a 70-day feeding trial using the recirculating aquacultural facility of Guangxi Minzu University (Nanning, Guangxi, China). The Experimental Animal Ethics Committee at Guangxi Minzu University (approval number 2024-016) reviewed and approved all animal-related protocols, including fish transport, acclimation, feeding management, and sample collection.

Before the trial, culture tanks were cleaned and disinfected with 10 mg/L potassium permanganate. Fish were also immersed in 10 mg/L potassium permanganate solution for 5 min before stocking. After disinfection, the fish were transferred to the recirculating aquaculture system and acclimated for 14 days. Throughout the acclimation phase, water temperature was held constant at 26 ± 0.5 °C, while dissolved oxygen was maintained above 7.0 mg/L, pH was adjusted to range between 7.0 and 8.5, total ammonia nitrogen was restricted to below 0.20 mg/L, and nitrite levels were kept under 0.10 mg/L. A 12L:12D photoperiod was adopted, with the photophase commencing at 08:00 and the scotophase concluding at 20:00. The basal diet was supplied to fish on three occasions each day (09:00, 14:00, and 19:00) until visual satiety was achieved.

A total of 360 juvenile largemouth bass were initially obtained for acclimation. After the acclimation period, 240 healthy fish with normal swimming behavior, active feeding, uniform body coloration, and similar body weight were selected for the feeding trial. Their initial body weight was 5.68 ± 0.46 g. Experimental fish were randomly distributed into four dietary groups (G0, G1, G2, and G3), with each group comprising three replicate tanks stocked with 20 individuals, yielding 12 tanks overall (1.2 m × 0.3 m × 0.6 m). The effective water volume during the experiment was approximately 180 L per tank, as the tanks were not completely filled to prevent fish from escaping and to accommodate the water circulation system. During the 70-day feeding period, fish in the four groups were fed diets containing 0, 200, 400, or 600 mg/kg CGA, respectively. Culture conditions were maintained as described for the acclimation period. Fish were fed three times daily at 09:00, 14:00, and 19:00, and the daily feeding rate was adjusted to approximately 5% of body weight. The recirculating system was supplied with uninterrupted aeration, while approximately 33% of the total water volume was replaced each day. Throughout the experimental period, fish health status, swimming activity, feed consumption, and survival were closely observed. Body weights were recorded biweekly, and the feeding ration was modified accordingly.

### 2.4. Sample Collection

Prior to sampling at the conclusion of the feeding trial, all fish were fasted for 24 h. From each tank within every treatment group, we randomly chose fish and subsequently immobilized them using MS-222 at 100 mg per liter (Adamas Reagent, Shanghai, China) before dissection. For biochemical assays and gene expression profiling, whole-intestine samples were taken from three fish per tank. These samples underwent gentle rinsing with ice-cold physiological saline for removal of intestinal debris, were blotted dry, promptly snap-frozen in liquid nitrogen, and stored at −80 °C until further processing. To analyze gut microbiota, intestinal samples collected from three individuals per tank were combined into a single biological replicate, rapidly snap-frozen via direct immersion into liquid nitrogen, then kept at minus 80 degrees Celsius. For histological assessment, foregut and midgut tissues were collected from three fish per tank. After careful removal of adhering fat and residual matter, these tissues were fixed in 4% paraformaldehyde for later histological examination. It is important to note that distinct fish were used for each type of analysis. Specifically, three fish per tank were used for whole-intestine tissue collection (for biochemical and gene expression analyses), three different fish per tank were used for histological examination (foregut and midgut), and another three separate fish per tank were used for intestinal content collection (microbiota analysis). No individual fish was subjected to more than one kind of analysis.

### 2.5. Determination of Intestinal Antioxidant-Related Parameters

Intestinal antioxidant-related indices were measured using commercial assay kits obtained from Nanjing Jiancheng Bioengineering Institute (Nanjing, China), and all assays were conducted according to the manufacturers’ protocols. Sample preparation was performed with reference to Li et al. [24], with minor modifications based on the characteristics of the intestinal tissue used in this study. The analyzed parameters included total antioxidant capacity (T-AOC; Cat. No. A015-2-1, 96T), superoxide dismutase (SOD; Cat. No. A001-2-2, 48T), catalase (CAT; Cat. No. A007-1-1, 96T), glutathione peroxidase (GSH-Px; Cat. No. A005-1-2, 48T), glutathione reductase (GR; Cat. No. A062-2-1, 96T), and malondialdehyde (MDA; Cat. No. A003-1-2, 96T). Whole-intestine samples were prepared according to Zhang et al. [25]: they were excised, rinsed with ice-cold physiological saline to clear intestinal contents, blotted dry, and weighed. For homogenization, ice-cold buffer was added at a 1:9 (*w*/*v*) ratio (tissue to buffer). The mixture was homogenized on ice and then centrifuged at 3000 rpm for 10 min at 4 °C. The resulting supernatant was collected for subsequent biochemical assays. Total protein content was concurrently measured and used to normalize enzyme activities and metabolite levels. T-AOC was quantified through the ABTS radical cation bleaching technique, with resultant values reported in U/mg protein. SOD activity was determined through a WST-1-based analytical assay. A single activity unit represented the quantity of enzyme capable of producing a 50% suppression of the reaction signal, and the final values were expressed as U/mg protein. The measurement of CAT activity utilized an ammonium molybdate-based colorimetric protocol. One activity unit was defined as the amount of enzyme required to degrade 1 μmol hydrogen peroxide within 1 s per milligram protein. GSH-Px and GR activities were both determined through colorimetric assays, with all quantification procedures and unit calibrations performed according to the manufacturer’s instructions. Values for GSH-Px were reported in U/mg protein, while GR values were given in U/g protein. MDA concentrations were determined via the thiobarbituric acid (TBA) color reaction assay, and the obtained values were expressed in nmol/mg protein.

### 2.6. Determination of Intestinal Gene Expression

We evaluated intestinal gene expression related to antioxidant defense, inflammation, and apoptosis following the method of Zhang and colleagues [26]. The genes examined included those related to immunity and inflammation, namely interleukin-1 beta (*il-1β*), interleukin-6 (*il-6*), tumor necrosis factor-alpha (*tnf-α*), interleukin-8 (*il-8*), interleukin-10 (*il-10*), nuclear factor kappa-B (*nf-κb*), nuclear factor kappa-B p50 subunit (*p50*), mitogen-activated protein kinase kinase kinase (*map3k*), Janus kinase 2 (*jak2*), signal transducer and activator of transcription 3 (*stat3*), transforming growth factor-beta (*tgf-β*); antioxidant-related genes, including catalase (*cat*), glutathione peroxidase (*gsh-px*), glutathione S-transferase (*gst*), superoxide dismutase (*sod*), nuclear factor erythroid 2-related factor 2 (*nrf2*), uncoupling protein 2 (*ucp2*), Kelch-like ECH-associated protein 1 (*keap1*); and cell injury- and apoptosis-related genes, including Bcl-2-associated X protein (*bax*), caspase-10 (*casp-10*), caspase-8 (*casp-8*), tumor protein p53 (*p53*), B-cell lymphoma 2 (*bcl2*), Bcl-2-associated athanogene (*bag*). We assessed the stability of two candidate housekeeping genes, *β-actin* and *ef1α*, with geNorm software (version 3.5). Both genes showed M values under the stability threshold (M < 0.5), with β-actin at 0.42 and ef1α at 0.38, suggesting high expression stability across all experimental groups. Therefore, they were chosen as reference genes for normalization, and the geometric mean of their Ct values was employed for relative quantification. The pairwise variation value V2/3 was 0.12, which is below the recommended cutoff of 0.15, confirming that the combination of these two reference genes was adequate for accurate normalization. All oligonucleotide primers were constructed based on publicly accessible largemouth bass genome or transcriptome data retrieved from the NCBI database, and were synthesized at Sangon Biotech Co., Ltd. (Shanghai, China). The corresponding sequences appear in Table 2.

Total RNA was isolated from whole-intestine samples using RNAiso Plus (TaKaRa Biotechnology, Dalian, China) following the manufacturer’s protocol. Isolated RNA was quantified and qualified using a NanoDrop 2000 spectrophotometer (Thermo Scientific, Waltham, MA, USA), with the A260/A280 absorbance ratio being documented. Furthermore, RNA intactness was validated by 1% agarose gel electrophoresis.

For first-strand cDNA synthesis, 1 μg of total RNA was treated with PrimeScript™ RT Reagent Kit with gDNA Eraser (TaKaRa Biotechnology, Dalian, China) to remove genomic DNA and perform reverse transcription according to the manufacturer’s instructions. The resulting cDNA was subsequently diluted 10-fold (1:10) with nuclease-free water and then employed for quantitative real-time PCR (qRT-PCR).

qRT-PCR was conducted using TB Green^®^ Premix Ex Taq™ II (Tli RNaseH Plus) (TaKaRa Biotechnology, Dalian, China) on a QuantStudio™ 5 Real-Time PCR System (Applied Biosystems, Waltham, MA, USA). Each reaction mixture had a final volume of 20 μL and contained 10 μL of 2× TB Green Premix, 0.4 μL of forward primer (10 μM), 0.4 μL of reverse primer (10 μM), 2 μL of cDNA template, and nuclease-free water to volume. The cycling regimen opened with a 30 s denaturation at 95 °C, then advanced through 40 iterations, each composed of a 5 s denaturation at 95 °C and a 30 s interval at 60 °C. Upon completion of the reaction, a dissociation curve analysis was conducted from 65 °C to 95 °C, with the temperature increasing by 0.5 °C at each 5 s interval. Specificity of amplification was verified by the appearance of a single melting-curve peak and the absence of obvious primer-dimer signals. We ran each sample in triplicate and incorporated a non-template control (NTC) into every assay. Relative transcript levels were determined via the 2^−ΔΔCt^ approach [27], using *β-actin* and *ef1α* as reference genes. The geometric mean of *β-actin* and *ef1α* was employed for normalization. Primer amplification efficiencies ranged from 96.8% to 106.1%, and correlation coefficients (R^2^) ranged from 0.991 to 0.999.

### 2.7. Intestinal Histological Observation

Intestinal histology was examined with reference to the method reported by Su et al. [28]. For histological analysis, foregut and midgut tissues were collected from fish of each treatment group. After dissection, adhering fat and residual intestinal contents were carefully removed, and the tissues were immediately immersed in 4% paraformaldehyde for fixation. The fixed samples were then submitted to a commercial laboratory (Servicebio Technology Co., Ltd., Wuhan, China) for conventional embedding in wax and HE staining. The processing sequence comprised dehydration, clearing, paraffin embedding, sectioning, staining, and mounting.

The stained sections were scanned into whole-slide images using a digital pathology scanner (PANNORAMIC DESK/MIDI/250/1000, 3DHISTECH, Budapest, Hungary). Image analysis of the foregut and midgut was carried out with CaseViewer software (CaseViewer 2.4, 3DHISTECH, Budapest, Hungary). Histomorphometric evaluation included villus length (abbreviated as VL), villus width (VW), as well as muscularis thickness (MT). All measurements were carried out in a blinded fashion, without knowledge of the treatment group assignments. For each intestinal segment of each sample, two complete microscopic fields were randomly chosen, and three intact villi were measured within each field. The average value from the selected fields was taken as the final measurement for that intestinal segment in each sample.

### 2.8. Intestinal Microbial Community Sequencing

Intestinal microbial community analysis was conducted following previously described procedures with minor modifications [29]. Immediately after collection, one whole-intestinal content sample from each replicate tank was snap-frozen in liquid nitrogen and subsequently shipped to Majorbio Bio-Pharm Technology Co., Ltd. (Shanghai, China) for sequencing analysis. Genomic DNA extraction, quality control, PCR amplification, library preparation, and high-throughput sequencing were all completed by the company according to standardized procedures. Microbial DNA was isolated with a commercially available bacterial DNA purification kit following the supplier’s recommended instructions. The bacterial 16S rRNA V3–V4 hypervariable fragments were subsequently amplified with primers 338F (5′-ACTCCTACGGGAGGCAGCAG-3′) and 806R (5′-GGACTACHVGGGTWTCTAAT-3′). PCR reactions were initiated with denaturation at 95 °C for 180 s, followed by 27 amplification rounds consisting of denaturation at 95 °C for 30 s, annealing at 55 °C for 30 s, and elongation at 72 °C for 45 s, and completed with a terminal extension step at 72 °C for 600 s. Sequencing libraries were prepared according to the standard protocol of Majorbio Bio-Pharm Technology Co., Ltd. (Shanghai, China). Paired-end sequencing of the amplified libraries was performed on an Illumina platform (Illumina, Inc., San Diego, CA, USA) according to the standard procedures of Majorbio Bio-Pharm Technology Co., Ltd. (Shanghai, China).

Raw reads were quality-filtered using fastp (version 0.23.4) and merged using FLASH (version 1.2.11). After quality control and merging, a total of 1,442,774 clean reads were obtained from the 12 samples (four dietary groups × three replicate tanks, one sample per tank), with an average read length of 423 bp. Operational taxonomic units (OTUs) were clustered at 97% sequence similarity using USEARCH (version 11), and chimeric sequences were removed, yielding 1414 OTUs. Taxonomic annotation was performed using the RDP Classifier (version 2.11) against the SILVA reference database (version 138) with a confidence threshold of 70%. To minimize the effect of sequencing depth, all samples were rarefied to 43,800 reads per sample.

Alpha diversity (Chao1, ACE, Shannon, and Simpson indices) and beta diversity (Bray–Curtis distance-based principal coordinates analysis, PCoA) were computed with Mothur (version 1.30.2), and differences in beta diversity among groups were tested by PERMANOVA. Taxonomic composition at phylum and order levels was analyzed and visualized using standard procedures implemented in the Majorbio Cloud Platform. In addition, the potential functional profiles of the intestinal microbiota were inferred using PICRUSt2 (version 2.2.0), and functional annotation was performed based on KEGG pathway annotation.

### 2.9. Statistical Analysis

Statistical computations were carried out with IBM SPSS Statistics 25 (IBM Corp., Armonk, NY, USA). Prior to statistical analysis, data were tested for normality using the Shapiro–Wilk test and for homogeneity of variance using Levene’s test. When the assumptions of normality and homogeneity of variance were satisfied, we compared treatment groups using one-way ANOVA, with Tukey’s HSD post hoc test for multiple comparisons. For data that did not satisfy the assumptions of normality or homogeneity of variance, non-parametric analyses were conducted using the Kruskal–Wallis H test followed by Dunn’s multiple comparison test. Because the PICRUSt2-predicted functional abundance data did not satisfy parametric assumptions, these data were analyzed using the same non-parametric procedures.

In the present study, each tank was considered the experimental unit, and three replicate tanks were included for each dietary treatment (*n* = 3). For biochemical parameters, gene expression analyses, and histological measurements, three fish were randomly sampled from each tank, and the mean value was used as the representative value for statistical analysis. For intestinal microbial community analysis, one whole-intestinal content sample from each tank was used as one biological replicate for statistical evaluation. Results are expressed as mean ± standard deviation (SD).

## 3. Results

### 3.1. Effects of Dietary Chlorogenic Acid Supplementation on Intestinal Antioxidant Capacity in Largemouth Bass

According to Table 3, the activities of T-AOC, SOD, CAT, GSH-Px, and GR were significantly higher in the G1, G2, and G3 groups compared to the G0 group (*p* < 0.05). By contrast, every CGA-supplemented group exhibited a marked reduction in MDA content relative to the control group (*p* < 0.05).

**Figure 1:**
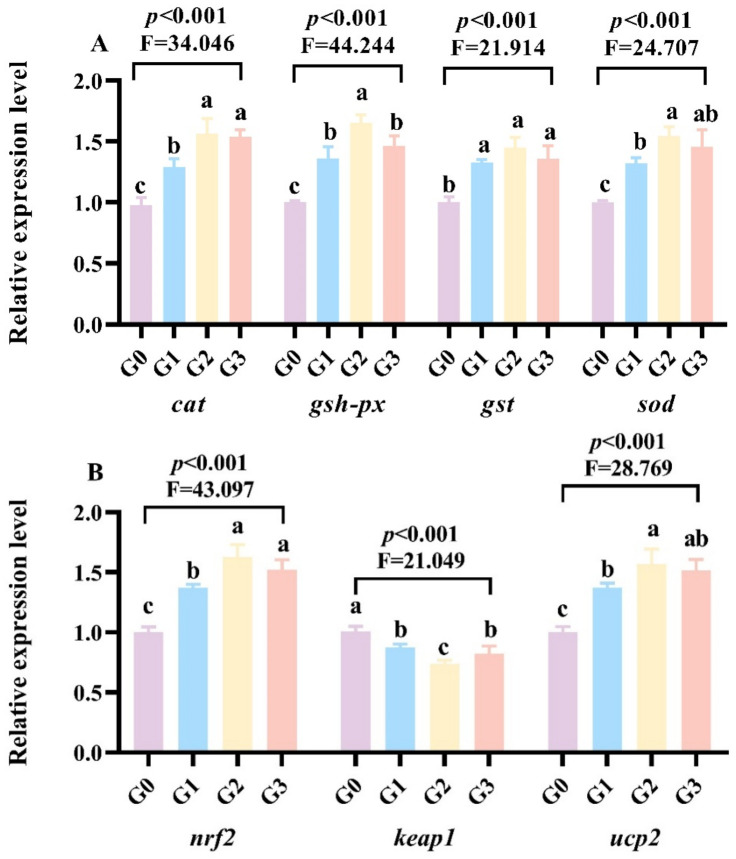
Effects of dietary chlorogenic acid supplementation on the expression of intestinal antioxidant-related genes in largemouth bass. (**A**) *cat*: Catalase; *gsh-px*: Glutathione peroxidase; *gst*: Glutathione S-transferase; *sod*: Superoxide dismutase. (**B**) *nrf2*: Nuclear factor erythroid 2-related factor 2; *keap1*: Kelch-like ECH-associated protein 1; *ucp2*: Uncoupling protein 2. Data are presented as mean ± SD (*n* = 3 tanks per treatment). In the same row, data with distinct superscript letters signify significant differences (*p* < 0.05).

The gene expression results showed a similar pattern. Compared with the G0 group, intestinal transcript levels of *cat*, *gsh-px*, *gst*, *sod*, *nrf2*, and *ucp2* were significantly elevated in the G1, G2, and G3 groups, whereas *keap1* expression was markedly reduced (*p* < 0.05; Figure 1).

### 3.2. Effects of Dietary Chlorogenic Acid Supplementation on Intestinal Inflammatory Responses in Largemouth Bass

**Figure 2:**
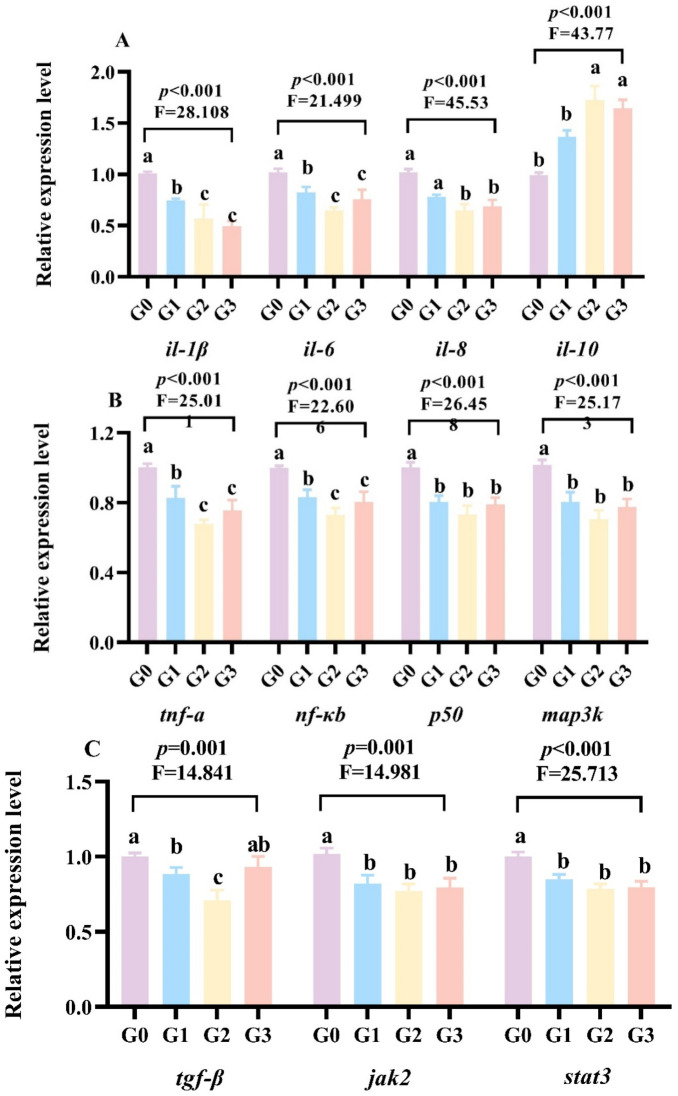
Effects of dietary chlorogenic acid supplementation on the relative expression of intestinal inflammation-related genes in largemouth bass. (**A**) *il-1β*: Interleukin-1 beta; *il-6*: Interleukin-6; *il-8*: Interleukin-8; *il-10*: Interleukin-10. (**B**) *tnf-α*: Tumor necrosis factor alpha; *nf-κb*: Nuclear factor kappa-B; *p50*: Nuclear factor kappa-B p50 subunit; *map3k*: Mitogen-activated protein kinase kinase kinase. (**C**) *tgf-β*: Transforming growth factor beta; *jak2*: Janus kinase 2; *stat3*: Signal transducer and activator of transcription 3. Data are presented as mean ± SD (*n* = 3 tanks per treatment). In the same row, data with distinct superscript letters signify significant differences (*p* < 0.05).

As shown in Figure 2, the intestinal expression of *il-1β*, *il-6*, *il-8*, *tnf-α*, *nf-κb*, *p50*, *map3k*, *jak2*, and *stat3* was markedly lower in the G1, G2, and G3 groups than in the G0 group (*p* < 0.05).

Additionally, a marked reduction in intestinal *tgf-β* expression was observed in the G1 and G2 groups versus the G0 group (*p* < 0.05). In contrast, all three CGA-supplemented groups exhibited a significant increase in *il-10* expression when compared with the control group (*p* < 0.05; Figure 2).

### 3.3. Effects of Dietary Chlorogenic Acid Supplementation on the Expression of Intestinal Apoptosis-Related Genes in Largemouth Bass

**Figure 3:**
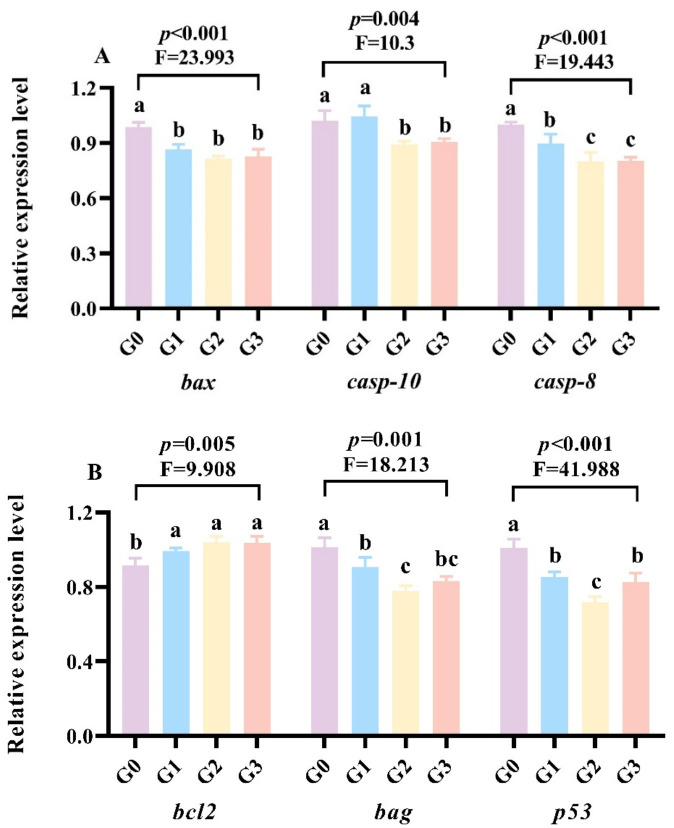
Effects of dietary chlorogenic acid supplementation on the relative expression of intestinal apoptosis-related genes in largemouth bass. (**A**) *bax*: Bcl-2-associated X protein; *casp-10*: Caspase-10; *casp-8*: Caspase-8. (**B**) *bcl2*: B-cell lymphoma 2; *bag*: Bcl-2-associated athanogene; *p53*: Tumor protein p53. Data are presented as mean ± SD (*n* = 3 tanks per treatment). In the same row, data with distinct superscript letters signify significant differences (*p* < 0.05).

Figure 3 shows that the intestinal expression levels of *bax*, *casp-8*, *p53*, and *bag* were significantly reduced in the G1, G2, and G3 groups compared with those in the G0 group (*p* < 0.05), whereas *bcl2* expression was substantially upregulated.

Moreover, a significant decrease in *casp-10* relative expression was detected in the G2 and G3 groups versus the G0 group (*p* < 0.05; Figure 3).

### 3.4. Effects of Dietary Chlorogenic Acid Supplementation on Intestinal Histomorphology in Largemouth Bass

**Figure 4:**
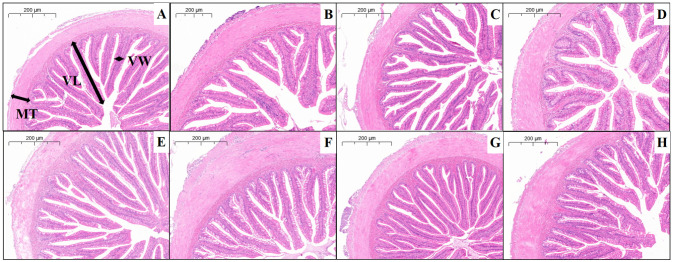
Effects of dietary chlorogenic acid supplementation on intestinal morphology in largemouth bass. (**A**), foregut of G0; (**B**), midgut of G0; (**C**), foregut of G1; (**D**), midgut of G1; (**E**), foregut of G2; (**F**), midgut of G2; (**G**), foregut of G3; (**H**), midgut of G3. MT, muscularis thickness; VL, villus length; VW, villus width. Hematoxylin and eosin (HE) staining. Scale bars = 200 μm.

The histological features and quantitative histomorphometric data are shown in Figure 4 and Table 4. In the foregut, muscularis thickness and villus length were significantly higher in the G1 and G2 groups than in the G0 group (*p* < 0.05), whereas villus width was significantly increased in all CGA-supplemented groups (*p* < 0.05). In the midgut, the G2 group showed the greatest muscularis thickness among all groups (*p* < 0.05). Villus length in the midgut proved markedly greater in the G2 and G3 groups than in the G0 group (*p* < 0.05). For villus width, the highest value was observed in the G2 group, while the G3 group did not differ significantly from the control group. Taken together, intestinal structural modifications varied across gut segments and supplementation doses, with the most conspicuous alterations being chiefly detected in the G2 group.

**Table 4:** Effects of chlorogenic acid supplementation in the diet on intestinal histomorphometry parameters in largemouth bass.

Index	Group			
	G0	G1	G2	G3
MT (μm)				
Foregut	206.2 ± 6.56 ^c^	232.47 ± 8.72 ^b^	262.47 ± 16.4 ^a^	225.27 ± 8.98 ^b,c^
Midgut	170.03 ± 11.19 ^b^	204.4 ± 4.84 ^b^	336.8 ± 33.11 ^a^	173.93 ± 11.93 ^b^
VL (μm)				
Foregut	646.3 ± 27.21 ^c^	761.17 ± 13.03 ^b^	924.07 ± 70.27 ^a^	728.4 ± 45.92 ^b,c^
Midgut	568.13 ± 29.39 ^c^	546.83 ± 7.44 ^c^	839.43 ± 25.55 ^a^	716.07 ± 36.3 ^b^
VW (μm)				
Foregut	83.7 ± 5.25 ^b^	118.17 ± 10.73 ^a^	115.7 ± 13.58 ^a^	108.4 ± 8.94 ^a^
Midgut	86.63 ± 2.15 ^c^	106.57 ± 4.08 ^b^	118.93 ± 10 ^a^	92.73 ± 6.45 ^c^

Note: Data are presented as mean ± SD (*n* = 3 tanks per treatment). In the same row, data with distinct superscript letters signify significant differences (*p* < 0.05). MT: Muscularis thickness; VL: Villus length; VW: Villus width.

### 3.5. Effects of Dietary Chlorogenic Acid Supplementation on Intestinal Microbial Diversity and Community Structure in Largemouth Bass

**Figure 5:**
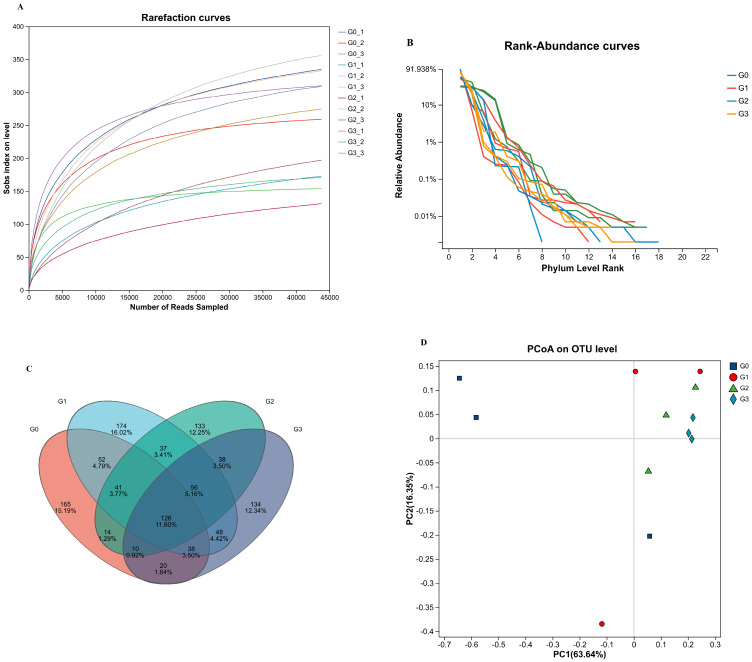
Diversity and community structure analysis of the intestinal microbiota in largemouth bass. (**A**) Rarefaction curves. (**B**) Rank abundance curves. (**C**) Venn diagram of shared and unique OTUs among different groups. (**D**) Principal coordinates analysis (PCoA) of intestinal microbial communities.

The rarefaction curves and rank abundance curves for all groups gradually approached a plateau with increasing sequencing depth, as shown in Figure 5 and Table 5. Moreover, Good’s coverage values exceeded 0.999 across all groups, confirming that the sequencing depth was adequate to capture the intestinal microbial composition of largemouth bass.

**Table 5:** Alpha diversity outcomes of the intestinal microbiota.

Index	Group			
	G0	G1	G2	G3
Shannon index	1.73 ± 0.16 ^c^	2.2 ± 0.15 ^a,b^	2.59 ± 0.33 ^a^	2.04 ± 0.22 ^b,c^
Simpson index	0.15 ± 0.11	0.43 ± 0.37	0.55 ± 0.17	0.57 ± 0.06
ACE index	269.32 ± 111.54	305.14 ± 79.66	298.17 ± 106.56	286.29 ± 84.42
Chao index	268.33 ± 110.08	304.33 ± 82.16	300.13 ± 102.8	297.22 ± 69.2
Coverage	0.9997 ± 0.0001 ^a^	0.9995 ± 0.0001 ^a,b^	0.999 ± 0.00022 ^b,c^	0.9993 ± 0.00012 ^c^

Note: Data are presented as mean ± SD (*n* = 3 tanks per treatment). In the same row, data with distinct superscript letters signify significant differences (*p* < 0.05).

Venn diagram analysis revealed that the G0, G1, G2, and G3 groups contained 165, 174, 133, and 134 unique OTUs, respectively, while 126 OTUs were found in every group (Figure 5). The occurrence of group-specific OTUs may imply that dietary CGA affected the composition of certain low-abundance intestinal microbial taxa, though the ecological roles of these microorganisms remain unclear.

Alpha diversity assessment showed that the Shannon index was markedly elevated in the G1 and G2 groups than in the G0 group (*p* < 0.05), with the highest value observed in the G2 group. The Simpson indices of the G2 and G3 groups were also elevated relative to the G0 group, albeit not significantly. In contrast, the ACE and Chao indices showed only minor variations among groups, whereas Good’s coverage stayed high across all treatments (Table 5). These observations indicate that CGA supplementation primarily influenced community evenness and the distribution of dominant taxa, with a relatively limited effect on total richness.

Beta diversity analysis further revealed a distinct segregation of the G0 group from the CGA-supplemented groups on the two-dimensional PCoA plot. PC1 and PC2 accounted for 63.64% and 16.35% of the overall variation, respectively (Figure 5). This finding indicates that dietary CGA supplementation modified the global intestinal microbial community structure of largemouth bass.

### 3.6. Effects of Dietary Chlorogenic Acid Supplementation on Intestinal Microbial Composition and Functional Prediction in Largemouth Bass

**Figure 6:**
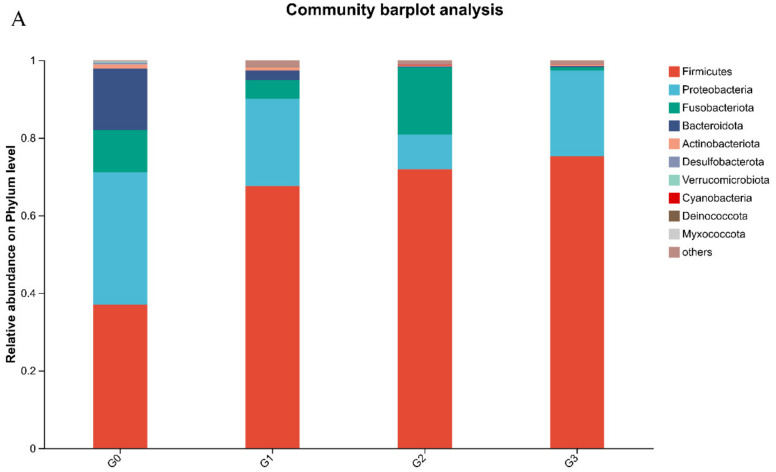

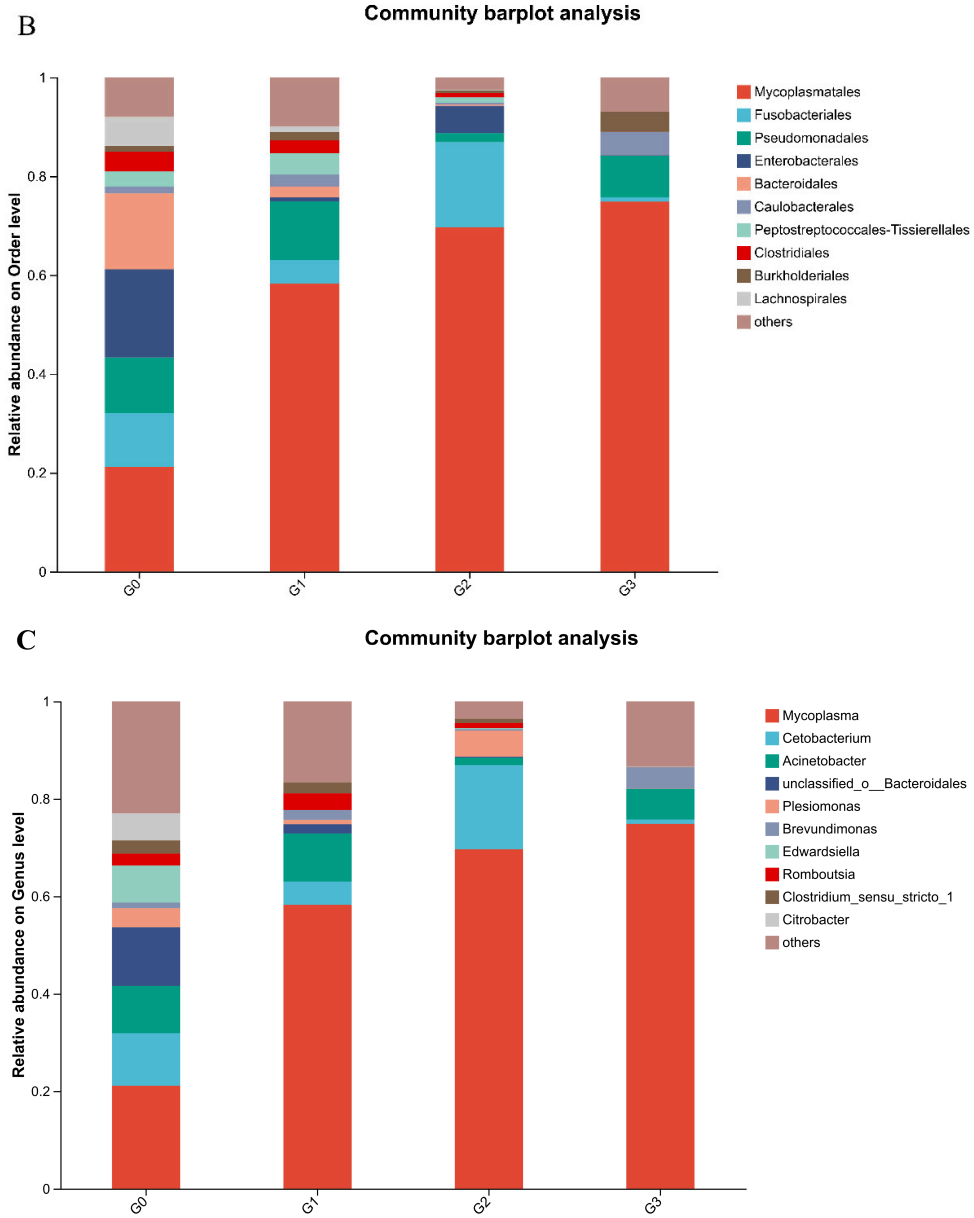

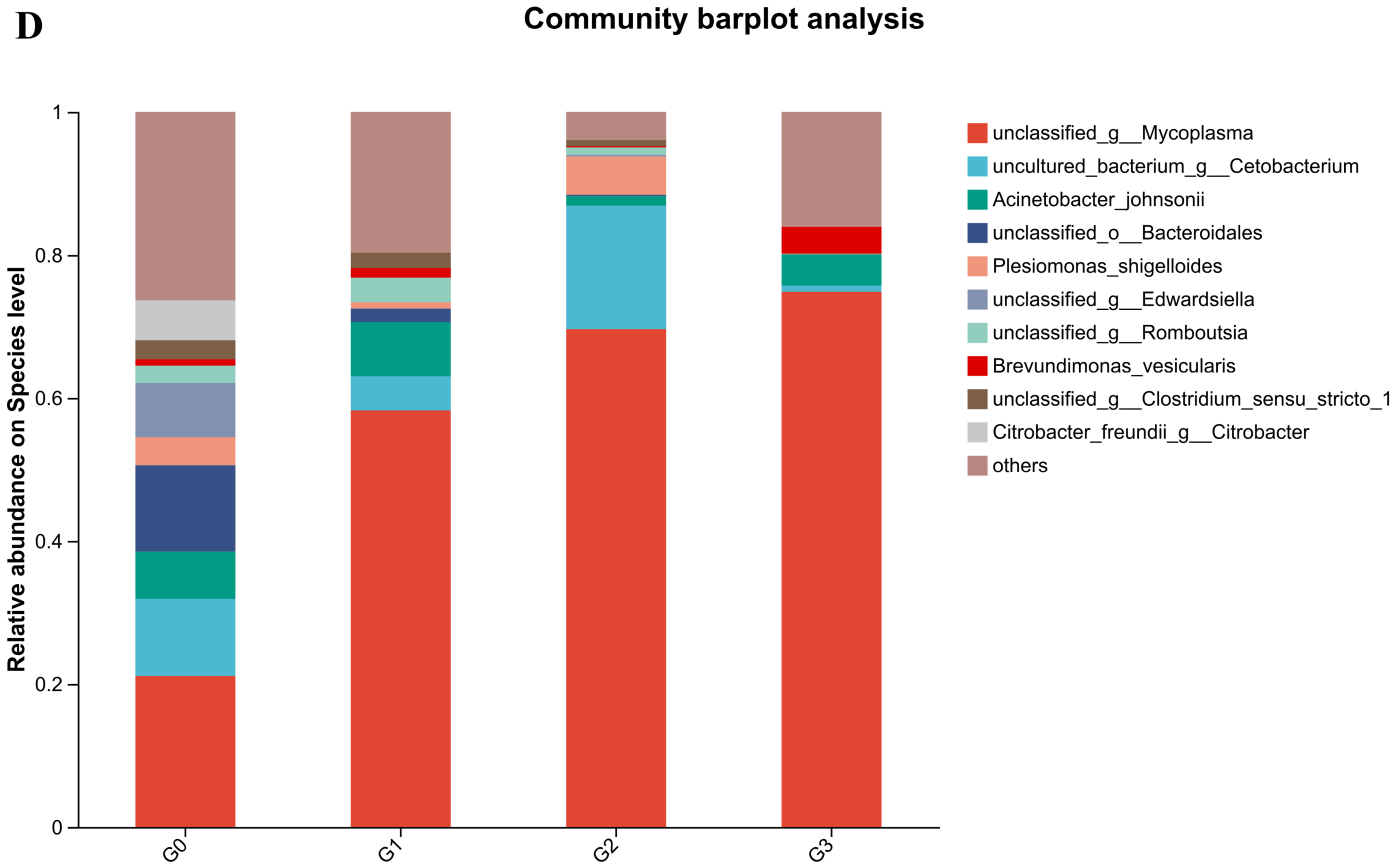
Relative abundance of intestinal microbiota at different taxonomic levels in largemouth bass. (**A**) Phylum-level composition. (**B**) Order-level composition. (**C**) Genus-level composition. (**D**) Species-level composition. Data are presented as descriptive relative abundances; formal differential abundance testing (e.g., LEfSe) was not performed due to limited sample size (*n* = 3 per group).

Figure 6A displays the phylum-level composition of the intestinal microbiota. In the control group (G0), the most abundant phyla were Firmicutes, Proteobacteria, and *Fusobacteriota*. Compared to G0, the CGA-supplemented groups (G1, G2, and G3) showed an increased abundance of *Firmicutes*, which became the dominant phylum, while Proteobacteria and *Fusobacteriota* both exhibited decreasing trends. Additionally, *Bacteroidota* was greatly reduced in the CGA-treated groups and was almost undetectable in certain samples.

At the order level, dietary CGA supplementation correlated with a rise in the relative abundance of *Mycoplasmatales*, with notably higher levels seen in the G2 and G3 groups. In contrast, the relative abundances of *Enterobacterales*, *Bacteroidales*, and several other orders generally decreased (Figure 6B).

Genus- and species-level analyses were further performed to refine the taxonomic interpretation of the intestinal microbiota (Figure 6C,D). CGA supplementation was associated with changes in several dominant lower-level taxa, including unclassified_g__Mycoplasma, uncultured_bacterium_g__Cetobacterium, Acinetobacter_johnsonii, and Plesiomonas_shigelloides. These results indicate that CGA altered the composition of dominant intestinal microbial taxa at lower taxonomic levels. However, these changes should be interpreted descriptively, as functional roles may differ among genera, species, and strains.

**Figure 7:**
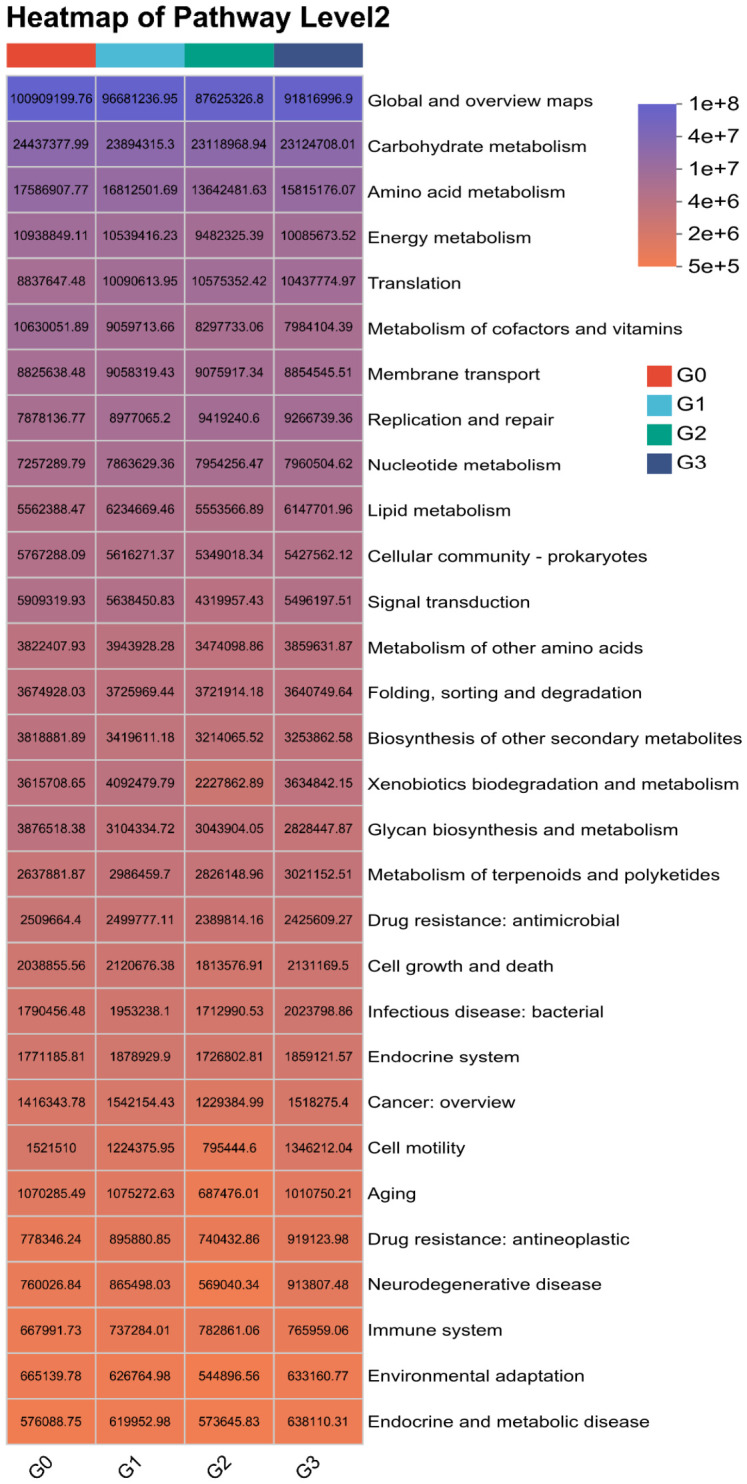
Heatmap of PICRUSt2-predicted functional abundance at KEGG pathway level 2 in the intestinal microbiota of largemouth bass.

According to the PICRUSt2 functional prediction heatmap, the CGA-treated groups exhibited generally higher predicted abundances than the G0 group in pathways involved in carbohydrate metabolism, amino acid metabolism, energy metabolism, lipid metabolism, membrane transport, as well as replication and repair. The G2 and G3 groups showed relatively greater predicted abundances for these pathways (Figure 7).

**Figure 8:**
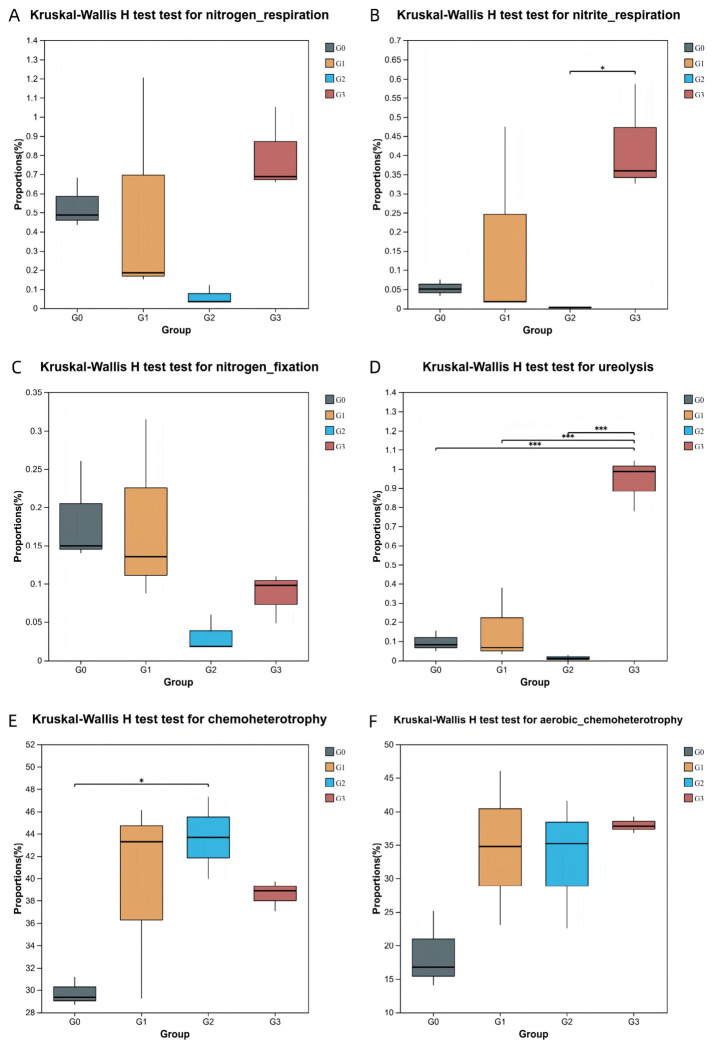

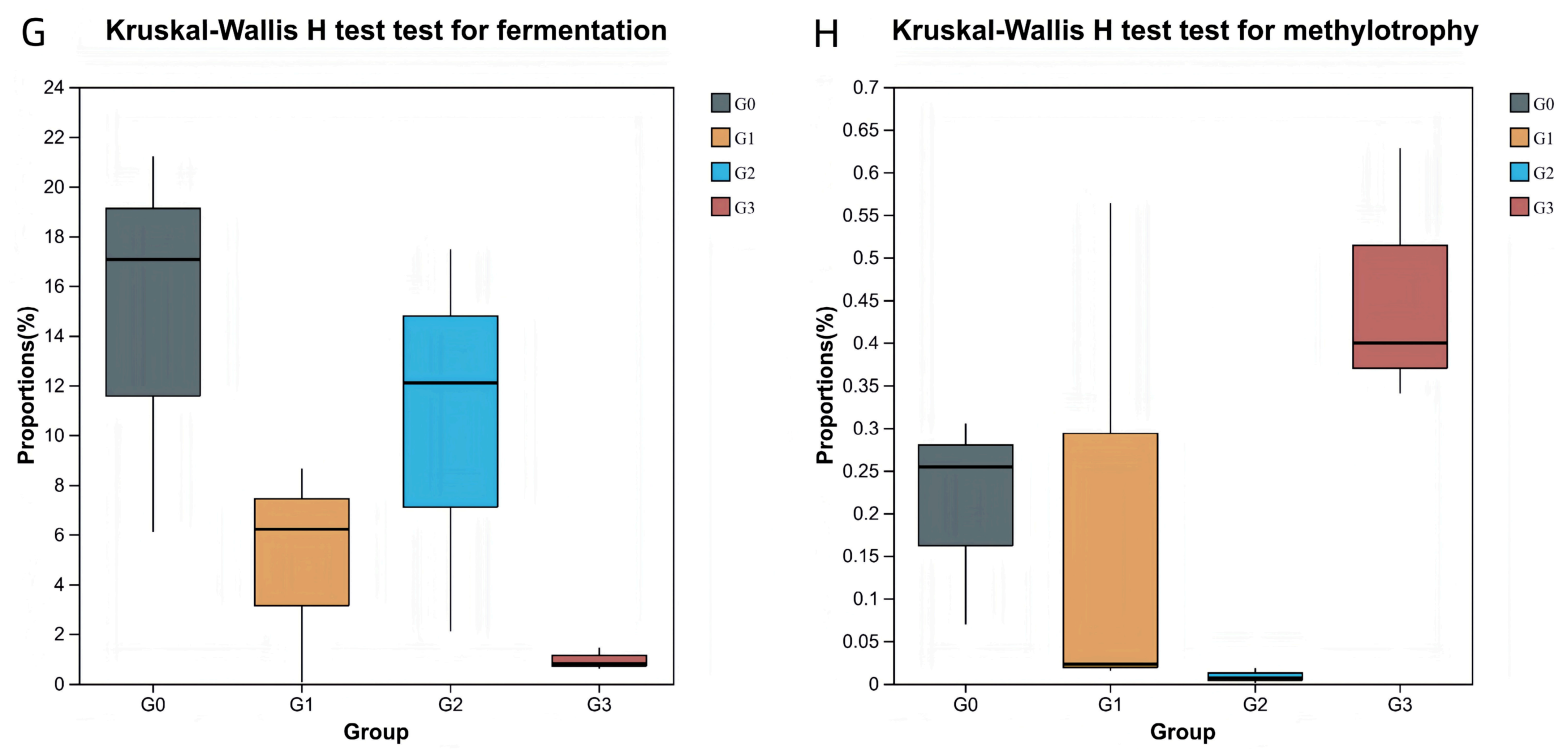
Kruskal–Wallis H test of selected level 3 functions predicted by PICRUSt2 in the intestinal microbiota of largemouth bass. (**A**–**D**) Nitrogen cycling-related functions, including nitrogen respiration, nitrite respiration, nitrogen fixation, and ureolysis. (**E**–**H**) Carbon source utilization and energy metabolism-related functions, including chemoheterotrophy, aerobic chemoheterotrophy, fermentation, and methylotrophy. * 0.01 < *p* ≤ 0.05; *** *p* ≤ 0.001.

Further analysis of selected level 3 functions using the Kruskal–Wallis H test showed that, among nitrogen cycle-related functions, nitrite respiration and ureolysis had relatively higher predicted abundances in the G3 group. Among functions related to carbon source utilization and energy metabolism, chemoheterotrophy and aerobic chemoheterotrophy exhibited higher levels in some chlorogenic acid-supplemented groups compared with the G0 group. Conversely, the G3 group exhibited lower fermentation levels but higher methylotrophy (*p* < 0.05) (Figure 8).

## 4. Discussion

### 4.1. Chlorogenic Acid Enhances Intestinal Antioxidant Defense in Largemouth Bass

Maintenance of intestinal redox homeostasis is fundamental to epithelial integrity and local physiological stability [30]. ROS overproduction can trigger lipid peroxidation along with cellular membrane damage. In this context, T-AOC, SOD, CAT, GSH-Px, and GR collectively indicate the total antioxidant defense capability of the organism, whereas MDA serves as a common marker for lipid peroxidation [31,32]. Here, dietary CGA supplementation resulted in elevated intestinal T-AOC and antioxidant enzyme activities, along with decreased MDA content, suggesting improved enzymatic antioxidant capacity and reduced lipid peroxidation within the intestine.

At the transcriptional level, dietary CGA upregulated the expression of *cat*, *gsh-px*, *gst*, *sod*, *nrf2*, and *ucp2*, while downregulating *keap1* expression. This expression profile is consistent with an enhanced antioxidant defense-related transcriptional pattern, suggesting that CGA may be involved in the regulation of intestinal antioxidant responses. The transcriptional changes observed in antioxidant-associated genes may be linked to stimulation of the Nrf2/Keap1 pathway, a key regulatory system responsible for preserving intracellular redox balance by controlling the expression of antioxidant defense enzymes [33]. Comparable findings were reported in juvenile spotted sea bass (*Lateolabrax maculatus*), where dietary CGA supplementation enhanced antioxidant enzyme activities and upregulated *nrf2*-related signaling, thereby alleviating oxidative stress-induced intestinal damage [34]. Previous investigations have indicated that CGA and other plant-derived polyphenolic compounds may confer cellular protection by neutralizing reactive oxygen species directly while simultaneously strengthening intrinsic antioxidant defense mechanisms [17,35,36]. Collectively, our observations align well with these previous reports and demonstrate that, even under unstressed farming conditions, dietary CGA can effectively enhance intestinal antioxidant capacity in largemouth bass.

In summary, the enzymatic and transcriptional results together suggest that one of the primary protective effects of CGA on the intestine is the reduction in oxidative damage. This improvement may further provide a physiological basis for subsequent regulation of inflammation-related responses and maintenance of intestinal tissue condition. However, because the present analyses were conducted primarily at the transcriptional level, additional protein-level investigations are needed to further clarify the involvement of the Nrf2/Keap1 pathway in CGA-mediated antioxidant regulation.

### 4.2. Chlorogenic Acid Reduces Inflammatory Burden and Improves Intestinal Tissue Condition

Inflammation-related transcriptional activity, indices of cellular injury, and histomorphological features together provide a comprehensive reflection of intestinal health [37,38]. In the present study, CGA decreased the expression of *il-1β*, *il-6*, *il-8*, *tnf-α*, *nf-κb*, *p50*, *map3k*, *jak2*, and *stat3*, while increasing *il-10* expression, indicating that CGA was associated with lower pro-inflammatory transcriptional activity and a relatively more stable local immune environment [16]. In addition, the reduced expression of pro-inflammatory cytokines may be associated with suppression of NF-κB-mediated inflammatory signaling, which is considered one of the major pathways involved in CGA-regulated anti-inflammatory responses in vertebrates [18]. In amur ide (*Leuciscus waleckii*), supplementation with chlorogenic acid in the diet alleviated intestinal oxidative injury, inflammatory responses, and apoptosis triggered by lipopolysaccharide exposure, while simultaneously suppressing the transcription of pro-inflammatory cytokines and inhibiting NF-κB-associated signaling pathways [39]. Moreover, beyond the alterations observed in pro-inflammatory cytokine expression, reduced *tgf-β* transcription was also detected in the G1 and G2 treatments. TGF-β is generally involved in mucosal immune regulation, epithelial maintenance, and local immune tolerance [40]. In teleosts, TGF-β is further recognized as an important mediator of adaptive mucosal immunity, contributing to epithelial immune tolerance and regulation of T-cell-associated immune functions [41]. A previous study in grass carp demonstrated that TGF-β can exert opposing effects on leukocytes, indicating a context-dependent regulatory function in teleost immunity [42]. Therefore, although the present study observed reduced *tgf-β* expression together with decreased pro-inflammatory transcriptional activity, the broader immunomodulatory roles of TGF-β in the fish intestine suggest that this transcriptional change should be interpreted within a more complex regulatory network. Thus, the reduction in *tgf-β* expression may indicate that CGA supplementation affected immune regulatory signaling in the intestine, together with its effects on other inflammation-related genes. These findings align well with earlier studies documenting the anti-inflammatory properties of CGA, and additionally imply that CGA might help sustain immune homeostasis within the intestinal tract of largemouth bass [22].

Regarding indicators associated with cellular injury, CGA modified the expression patterns of *bax*, *casp-8*, *casp-10*, *p53*, *bcl2*, and *bag*. Several pro-apoptotic genes showed significant downregulation, implying that intestinal cellular stress may have been reduced, whereas the anti-apoptotic gene *bcl2* was upregulated [43]. In contrast, *bag*, which encodes an anti-apoptotic co-chaperone involved in cellular stress regulation, also showed reduced expression. This transcriptional response may reflect alterations in intestinal cellular regulatory processes under CGA supplementation, although its precise functional significance requires further investigation. Functionally, *bax*, caspase family members, and *p53* are commonly linked to cellular stress responses and the initiation of apoptosis, whereas increased *bcl2* expression is generally considered favorable for cell survival and maintenance of mitochondrial membrane stability [39]. Whether these transcriptional changes are ultimately accompanied by measurable alterations in apoptotic phenotype still requires confirmation through further functional analyses. Accordingly, the present findings should be interpreted as transcriptional-level responses.

Histological findings provided additional evidence for the beneficial effects of CGA on intestinal condition. Compared with the control group, CGA supplementation increased villus length, villus width, and, in some intestinal segments, muscularis thickness in both the foregut and midgut, with the G2 group showing more evident changes in several histomorphological parameters. Improved villus architecture generally reflects an expansion of the absorptive surface of the mucosa, whereas greater muscularis development may contribute to support structural stability of the intestine [34,44]. Histomorphological improvements induced by dietary CGA were more evident in the G2 group, although the responses varied among intestinal segments. When considered together with the improvements in redox status and inflammation-related transcriptional patterns described above, these results suggest that CGA improved the intestinal microenvironment and tissue condition and may contribute to the maintenance of intestinal tissue condition and barrier function.

Taken together, the antioxidant, inflammation-related transcriptional, and histological results suggest that dietary CGA supplementation influenced intestinal physiological status in largemouth bass. Within the tested supplementation range, the 400 mg/kg group showed relatively favorable changes in several antioxidant, inflammation-related, and histomorphological indicators under the present experimental conditions.

### 4.3. Chlorogenic Acid Reshapes the Intestinal Microbial Community

The gut microbiota is closely related to nutrient utilization, local immune regulation, and intestinal tissue condition in fish [8]. In the present study, the rarefaction curves gradually approached saturation, and Good’s coverage values remained above 0.999, suggesting sufficient sequencing depth to characterize the dominant microbial taxa within the samples. According to alpha diversity analysis, CGA supplementation exerted minor influences on the ACE and Chao indices, while the G2 group displayed a higher Shannon index. This suggests that CGA mainly affected community diversity and evenness rather than simply increasing microbial richness. In addition, the PCoA results showed a separation between the control group and the CGA-supplemented groups, indicating that dietary CGA was associated with changes in intestinal microbial community structure.

At the phylum level, CGA supplementation was associated with an increase in Firmicutes and decreases in *Proteobacteria* and *Fusobacteriota*. These phylum-level shifts provide broad ecological information, but their functional implications require cautious interpretation because members within the same phylum may differ substantially in metabolic activity, ecological function, and host interactions. At the order level, the relative abundance of *Mycoplasmatales* increased in the CGA-supplemented groups, especially in the G2 and G3 groups, whereas *Enterobacterales*, *Bacteroidales*, and several other orders showed lower relative abundances. Previous fish microbiome studies have shown that members of *Mycoplasmatales* are commonly detected in the intestinal microbiota of several aquatic species and may participate in host–microbe interactions under specific intestinal environments. For example, Mycoplasma-dominated microbial communities have been reported in salmonids, where they are thought to contribute to intestinal microbial community assembly [45]. However, some *Mycoplasmatales* taxa have also been linked to opportunistic infection or host physiological disturbance in aquatic animals. Therefore, the ecological role of *Mycoplasmatales* in fish intestine may depend on host species, environmental conditions, and microbial strain composition. Here, the elevated abundance of *Mycoplasmatales* suggests that dietary CGA may have altered the ecological structure of the intestinal microbiota, though its functional relevance in largemouth bass remains to be further clarified.

At lower taxonomic levels, CGA supplementation was associated with changes in several dominant taxa, including unclassified_g__Mycoplasma, uncultured_bacterium_g__Cetobacterium, Acinetobacter johnsonii, and Plesiomonas shigelloides. These findings suggest that CGA-related microbial changes occurred not only at broad taxonomic levels but also among specific dominant taxa. Some of these taxa have previously been associated with nutrient metabolism, intestinal environmental adaptation, or opportunistic pathogenicity in aquatic animals, indicating that the microbial responses to dietary CGA may involve multiple ecological functions within the intestinal environment. Nevertheless, these results should be interpreted as compositional changes rather than direct functional evidence, because microbial functions may differ among genera, species, and strains. Further differential abundance analysis, bacterial isolation, and functional validation are needed to clarify the biological significance of these taxa.

PICRUSt2 analysis indicated potential functional differences in the intestinal microbiota among dietary groups [46]. Because this prediction is based on 16S rRNA profiles, the results should be regarded as inferred functional trends rather than direct measurements of microbial activity. Further multi-omics validation will help clarify the functional relevance of these predicted pathways. In the CGA-treated groups, particularly G2 and G3, higher predicted abundances were observed for carbohydrate-associated pathways, including amino acid, energy, and lipid metabolism, as well as replication and repair, and membrane transport. These findings imply that dietary CGA might affect the potential functional capacity of the gut microbial community, rather than providing direct evidence for enhanced nutrient metabolism or energy utilization.

At level 3, several predicted functions associated with nitrogen-related processes and carbon source utilization also differed among groups. Predicted ureolysis and some substrate utilization-related functions were relatively higher in the high-dose group, while predicted fermentation was lower and methylotrophy was higher. These results indicate that CGA supplementation may be linked to shifts in the potential metabolic profile of the intestinal microbiota. However, direct validation through metagenomic, transcriptomic, or metabolomic approaches is still needed to determine whether these predicted changes correspond to actual metabolic activity in vivo.

Taken together, dietary CGA supplementation was associated with changes in intestinal microbial diversity, dominant taxonomic composition, and predicted microbiota-derived functional profiles in largemouth bass. Across the evaluated treatments, the 400 mg/kg group exhibited elevated Shannon diversity and notable compositional shifts among several dominant taxa, suggesting that this concentration corresponded to microbial community alterations under the current experimental conditions. These microbial shifts, together with the antioxidant and histological responses observed in this study, suggest a possible link between dietary CGA and intestinal physiological status. Nevertheless, the functional relevance of the altered taxa and predicted pathways remains to be further confirmed using more direct approaches. In addition, the microbiota analysis was conducted using three biological replicates per treatment, which may have limited the statistical sensitivity for detecting subtle community differences, particularly in richness-related diversity indices. Therefore, the observed microbial variations should be interpreted as indicative ecological patterns under the present experimental conditions.

### 4.4. Integrated Interpretation of Intestinal Responses to Dietary Chlorogenic Acid

The present findings further suggest that the intestinal microbiota alterations induced by dietary CGA may be mechanistically linked to the observed modulation of inflammatory responses, oxidative status, and intestinal morphology. Intestinal microorganisms can regulate host redox and immune homeostasis via microbial metabolites, epithelial interactions, as well as modulation of signaling pathways linked to inflammation and oxidative stress [47]. Throughout host development, the gut microbiota and the mucosal immune system engage in constant crosstalk, thereby influencing the maturation of immunity, the integrity of the epithelium, and inflammatory reactions through metabolites and molecular structures derived from the microbiota [48]. In the present study, the increased abundance of potentially beneficial bacterial taxa together with the reduction in certain inflammation-associated microorganisms in the CGA-supplemented groups may have contributed to the inhibition of NF-κB-driven inflammatory signaling and the boost in antioxidant regulation associated with the Nrf2/Keap1 pathway. Reduced inflammatory responses and oxidative stress could subsequently favor the maintenance of intestinal epithelial integrity and villus structure, thereby contributing to improved intestinal health. Similar coordinated interactions among intestinal microbiota modulation, antioxidant regulation, inflammatory signaling, and intestinal barrier function have also been reported in fish receiving dietary functional additives, including chlorogenic acid and other plant-derived bioactive compounds. In common carp (*Cyprinus carpio*), for instance, dietary supplementation with chlorogenic acid has been demonstrated to enhance both intestinal immune condition and antioxidant capability via regulation of signaling pathways associated with NF-κB and Nrf2 [16]. Similarly, dietary phytogenic additives in several fish species have been linked to microbiota-mediated intestinal immune regulation. Nonetheless, this work mainly relied on correlative findings, and subsequent analyses at both the strain level and the mechanistic level will be required to elucidate the causal links between microbial shifts, redox modulation, and intestinal immune reactions in largemouth bass. It should also be noted that the biochemical, transcriptional, histological, and microbiota analyses were conducted using different intestinal regions or biological compartments, which may limit direct cross-comparison among datasets. Nevertheless, the broad consistency of the observed physiological responses supports the general interpretation of CGA-mediated intestinal regulation.

## 5. Conclusions

In conclusion, under the experimental conditions of this study, dietary chlorogenic acid supplementation improved the intestinal antioxidant capacity of largemouth bass, as reflected by enhanced antioxidant enzyme activities together with increased expression of antioxidant-related genes. Meanwhile, CGA reduced the expression of several inflammation-related genes, improved intestinal histomorphological characteristics, and modified gut microbial diversity, community structure, and the composition of major taxonomic groups. Taken together, these results suggest that CGA helps improve intestinal redox balance, inflammation-related transcriptional status, tissue condition, and gut microecology in largemouth bass. Within the tested supplementation range, the 400 mg/kg group showed relatively favorable changes in several intestinal indicators and may serve as a reference level for future studies evaluating dietary CGA supplementation in largemouth bass. Because the present study was conducted under normal culture conditions without pathogen challenge or environmental stress exposure, the observed responses mainly reflect baseline physiological regulation associated with dietary CGA supplementation. In addition, dietary CGA concentrations were not analytically verified after feed processing, and future studies integrating stress models and dietary compound stability analyses are needed to strengthen the understanding of CGA function in largemouth bass nutrition. Furthermore, the limited number of biological replicates (*n* = 3 per treatment) may affect the statistical robustness of some findings, particularly for microbiota and gene expression analyses; therefore, the results should be interpreted as preliminary, and future studies with larger sample sizes are warranted.

## Figures and Tables

**Table 1:** Composition of the experimental diets for juvenile largemouth bass (g/kg dry diet).

Ingredient	Group			
	G0	G1	G2	G3
Chlorogenic acid	0	0.2	0.4	0.6
Fish oil	30	30	30	30
Fish meal	490	490	490	490
Soybean meal	20	20	20	20
Soybean flour	235	235	235	235
Wheat flour	150	149.8	149.6	149.4
Yeast powder	30	30	30	30
Lecithin	10	10	10	10
Vitamin premix	6	6	6	6
Mineral premix	8	8	8	8
Choline chloride	6	6	6	6
Calcium phosphate	12	12	12	12
Antioxidant	3	3	3	3
Nutritional composition (% dry weight)				
Crude protein	46.91	46.91	46.91	46.91
Crude fat	11.35	11.35	11.35	11.35
Ash	9.36	9.36	9.36	9.36

Note: The vitamin premix provided the following per kilogram of diet: VA 18 mg, VD 3.5 mg, VE 150 mg, VC 150 mg, VB_1_ 16 mg, VB_6_ 20 mg, VB_12_ 18 mg, riboflavin 40 mg, inositol 320 mg, calcium pantothenate 60 mg, niacinamide 80 mg, folic acid 5 mg, biotin 2 mg, and ethoxyquin 100 mg. The mineral premix provided the following per kilogram of diet: Na 30 mg, K 50 mg, Mg 100 mg, Cu 4 mg, Fe 25 mg, Zn 35 mg, Mn 12 mg, I 1.6 mg, Se 0.2 mg, and Co 0.8 mg. CGA values indicate formulated supplementation levels.

**Table 2:** Real-time quantitative PCR primers used in this study.

Gene	Primer Sequence (5′–3′)	GenBank	Tm (°C)	Size (bp)	Efficiency (%)	R^2^
*β-actin* ^1^	F: GATGTGGATCAGCAAGCAGGAG	XM_038695351.1	60	117	97.8	0.998
	R: AGTCGTTTGGGTTTGTAGCAGTG					
*ef1α* ^2^	F: TGCTGCTGGTGTTGGTGAGTT	GU136229	60	147	105.4	0.995
	R: TTCTGGCTGTAAGGGGGCTC					
*il-1β* ^3^	F: ACTGGTGAATGTTGCTGTTGGAC	XM_038725717.1	60	123	100.5	0.997
	R: TCCTCCGTGGCTGATTTCTCTAC					
*il-6* ^4^	F: GGAACCCTGAACAGGTAACG	XM_038732985.1	60	100	99.1	0.996
	R: TGTGCGGTCATCTTTCTGTGG					
*tnf-α* ^5^	F: AGTGTTTGCTGGTTCTGAATGAAATG	XM_038723994.1	60	92	98.7	0.999
	R: CTTGCGGTACTGTGGCTTGAG					
*il-8* ^6^	F: CGTTGAACAGACTGGGAGAGATG	XM_038713528.1	60	81	102.3	0.994
	R: AGTGGGATGGCTTCATTATCTTGT					
*il-10* ^7^	F: TTAAGCCAGCAGCATCATTACCAC	XM_038696252.1	60	118	103.6	0.998
	R: ACCAGGACGGACAGGAGGAG					
*nf-κb* ^8^	F: AACAACAGCAACAACAGTCACAATAC	XM_038699446.1	60	114	99.8	0.996
	R: CCAGGATAACTAACTCGCTCAGATG					
*p50* ^9^	F: TGGACTCTGCTACTCTGGATCAAG	XM_038699792.1	60	93	104.2	0.993
	R: CTTCAATCGCTGCTTTGTGCTTAG					
*map3k* ^10^	F: GGAGGACGAGCACCGAGAC	XM_038737543.1	60	96	98.3	0.997
	R: CAAGGAGACCGTATTACACAGCAG					
*jak2* ^11^	F: TTTGGTGCTCTGCGTGTCTGTC	XM_038703932.1	60	138	98.5	0.995
	R: TGGTGTCCGAGTCCGATGTAGTG					
*stat3* ^12^	F: AGAACAATGTACTCTACCAGCATAACC	XM_038704527.1	60	84	101.1	0.999
	R: CAATGTCCATCGGCTTCTCCAAG					
*tgf-β* ^13^	F: GTATAAGCACCACAACCCAGGAG	XM_038720010.1	60	95	99.5	0.992
	R: GTTGCCTGCCCACGTAGTAAAG					
*cat* ^14^	F: TGGCTATGGCTCTCACACCTTC	XM_038704976.1	60	109	99.0	0.998
	R: CTCCTCTACTGGCAGATTCTTTATTCC					
*gsh-px* ^15^	F: AAAGTCTCTTAAGTATGTCCGTCCAG	XM_038736256.1	60	85	101.8	0.994
	R: ATCCTTTCCATTCACATCCACCTTC					
*gst* ^16^	F: ATGACGCCGCCGCTGAG	XM_038723659.1	60	119	98.9	0.997
	R: GAGTTGCTACGAGGATAACGAGTC					
*sod* ^17^	F: GCATCATAGGTCGCTCAGTTGTG	XM_038708943.1	60	99	97.2	0.996
	R: TGGCTCTGGCTACAGTCACTTC					
*nrf2* ^18^	F: TCCGCAAGAGGCAGCAGAC	XM_038697614.1	60	117	102.8	0.995
	R: GGTTGGGTTTGGATGGAGAGATAC					
*ucp2* ^19^	F: CCTGGGCTCCTGGAATGTGG	XM_038707996.1	60	97	97.5	0.993
	R: TGGTTAAGGTGTAGTTGTGTAGTTGTG					
*keap1* ^20^	F: TGTAATCACTGTATGGTCTGCTTCTTC	XM_038728590.1	60	110	100.0	0.998
	R: CTCAGGCTGGTTCATTGGTGTTC					
*bax* ^21^	F: TGTCGTCACCCGCCTCTTTC	XM_038704178.1	60	117	96.8	0.991
	R: ATAGCCAGTCTCTTCTTCTCTGTCTC					
*casp-10* ^22^	F: GCTTGGCTTTGAGGTAGTGATAGAG	XM_038720546.1	60	114	106.1	0.992
	R: ACGCAGCATACCAGGCAGTC					
*casp-8* ^23^	F: CACAACGTACCACCAGGATGAAG	XM_038726463.1	60	109	100.5	0.999
	R: TGTCCAGCAGTGACTCCAGAAC					
*p53* ^24^	F: AGGCAGGAATGACCAGTGGAAC	XM_038737455.1	60	120	103.6	0.996
	R: ACAATGGAGATGATGAAGGTGATGATG					
*bcl2* ^25^	F: CATCGGAGCATACCTAACACAGAAG	XM_038703623.1	60	84	102.3	0.997
	R: AGGAAGAGGAGGAGGAGGATGAG					
*bag* ^26^	F: CAGCAGCAGCAACAGCATCC	XM_038717684.1	60	106	98.7	0.994
	R: CCAGAGACGCCACCTCCTTC					

Note: F indicates forward primer; R indicates reverse primer. ^1^ *β-actin*: Reference gene 1. ^2^ *ef1α*: Reference gene 2. ^3^ *il-1β*: Interleukin-1 beta. ^4^ *il-6*: Interleukin-6. ^5^ *tnf-α*: Tumor necrosis factor alpha. ^6^ *il-8*: Interleukin-8. ^7^ *il-10*: Interleukin-10. ^8^ *nf-κb*: Nuclear factor kappa-B. ^9^ *p50*: Nuclear factor kappa-B p50 subunit. ^10^ *map3k*: Mitogen-activated protein kinase kinase kinase. ^11^ *jak2*: Janus kinase 2. ^12^ *stat3*: Signal transducer and activator of transcription 3. ^13^ *tgf-β*: Transforming growth factor beta. ^14^ *cat*: Catalase. ^15^ *gsh-px*: Glutathione peroxidase. ^16^ *gst*: Glutathione S-transferase. ^17^ *sod*: Superoxide dismutase. ^18^ *nrf2*: Nuclear factor erythroid 2-related factor 2. ^19^ *ucp2*: Uncoupling protein 2. ^20^ *keap1*: Kelch-like ECH-associated protein 1. ^21^ *bax*: Bcl-2-associated X protein. ^22^ *casp-10*: Caspase-10. ^23^ *casp-8*: Caspase-8. ^24^ *p53*: Tumor protein p53. ^25^ *bcl2*: B-cell lymphoma 2. ^26^ *bag*: Bcl-2-associated athanogene.

**Table 3:** Effects of dietary chlorogenic acid supplementation on intestinal antioxidant-related parameters in juvenile largemouth bass.

Index	Group			
	G0	G1	G2	G3
T-AOC ^1^ (U/mg prot)	0.61 ± 0.004 ^c^	0.73 ± 0.01 ^b^	0.85 ± 0.02 ^a^	0.87 ± 0.01 ^a^
SOD ^2^ (U/mg prot)	182.49 ± 6.28 ^c^	215.75 ± 6.72 ^b^	236.21 ± 11.01 ^a^	232.88 ± 2.69 ^a^
CAT ^3^ (U/mg prot)	14.08 ± 0.81 ^c^	18.97 ± 1.16 ^b^	23.71 ± 1.15 ^a^	22.38 ± 1.97 ^a^
MDA ^4^ (nmol/mg prot)	24.67 ± 0.77 ^a^	21.76 ± 0.97 ^b^	18.62 ± 0.83 ^c^	17.97 ± 1.07 ^c^
GSH-Px ^5^ (U/mg prot)	382.92 ± 7.74 ^c^	423.14 ± 12.46 ^b^	462.93 ± 11.54 ^a^	460.89 ± 11.19 ^a^
GR ^6^ (U/g prot)	18.67 ± 1.14 ^c^	24.3 ± 1.02 ^b^	32.94 ± 2.66 ^a^	32.58 ± 0.51 ^a^

Note: Data are presented as mean ± SD (*n* = 3 tanks per treatment). In the same row, data with distinct superscript letters signify significant differences (*p* < 0.05). ^1^ T-AOC: Total antioxidant capacity. ^2^ SOD: Superoxide dismutase. ^3^ CAT: Catalase. ^4^ MDA: Malondialdehyde. ^5^ GSH-Px: Glutathione peroxidase. ^6^ GR: Glutathione reductase.

## Data Availability

The data that support the findings of this study are available from the corresponding authors upon reasonable request.
